# Around the Hospitals

**Published:** 1894-03-10

**Authors:** 


### AROUND THE HOSPITALS.

Notice to the Managers of Institutions.—Special space is now reserved for the insertion of notices of hospital meetings and festivals. The Editor requests that all notices may reach The Hospital Office, 428, Strand, at least one week before the date of each meeting.

**The Hospital for Sick Children, Pendlebury.**—This hospital starts on the new year with the happy record that the burden of debt has been removed from its shoulders. Last year the balance against the hospital stood at upwards of £4,000. It is to the energy of the Mayor and Lady Mayoress that the prosperity is due. The latter called together a number of ladies, under whose auspices Manchester was divided into districts for systematic canvass, which resulted in a collection of £1,375. Later in the year the Mayor also convened a meeting to plead for contributions, whereby Mr. Agnew's generous offer of a donation of £500, should the remainder of the sum owing be forthcoming, could be claimed. That the whole amount was subscribed proves that the good work accomplished by the institution is appreciated, and that Manchester has only to awaken to the fact that funds are wanted. Annual subscriptions are the weak point in the finances, for they show very little growth, though the number of beds now maintained has risen from 25 to 140, and the reliable income of the hospital stands at £1,000 less than its needs. During the past year 1,332 cases have been received into the hospital, out-patients at the dispensary numbering 9,711. The cost of each in-patient averaged £5 11s. 8d., and that of out-patients averaged 1s. 7¾d. per head. Both these sums show a slight decrease as compared with the cost of the previous year. The convalescent funds of the institution are prospering, and it was possible to send 313 to convalescent homes during the year. It is felt that a permanent seaside home in connection with the hospital is much needed, and it is suggested that funds from the David Lewis Bequest might well be devoted to this purpose.

**Royal Southern Hospital, Liverpool.**—One feature of note in the report of this hospital's work during the past year is the success that has attended the experiment of establishing a ward for women sufferers of small means, but willing to pay a portion of the cost of their maintenance. It has been decided to open a like ward for men. The establishment of such wards in hospitals will tend to raise self-respect, and by the force of example much of the abuse of hospitals will be removed. The institution has received several substantia llegacies during the year. Some of these were for definite objects, and the remainder were required to meet current expenses. Although the income of the institution is still inadequate, the annual subscriptions show an upward movement. In-patients during the past year numbered 2,227, the out-patients 8,057, an increase on the figures of 1892.

